# Performance analysis and knowledge-based quality assurance of critical organ auto-segmentation for pediatric craniospinal irradiation

**DOI:** 10.1038/s41598-024-55015-7

**Published:** 2024-02-21

**Authors:** Emeline M. Hanna, Emma Sargent, Chia-ho Hua, Thomas E. Merchant, Ozgur Ates

**Affiliations:** https://ror.org/02r3e0967 grid.240871.8 0000 0001 0224 711X St. Jude Children’s Research Hospital, Memphis, TN 38105 USA

**Keywords:** Paediatric cancer, Radiotherapy

## Abstract

Craniospinal irradiation (CSI) is a vital therapeutic approach utilized for young patients suffering from central nervous system disorders such as medulloblastoma. The task of accurately outlining the treatment area is particularly time-consuming due to the presence of several sensitive organs at risk (OAR) that can be affected by radiation. This study aimed to assess two different methods for automating the segmentation process: an atlas technique and a deep learning neural network approach. Additionally, a novel method was devised to prospectively evaluate the accuracy of automated segmentation as a knowledge-based quality assurance (QA) tool. Involving a patient cohort of 100, ranging in ages from 2 to 25 years with a median age of 8, this study employed quantitative metrics centered around overlap and distance calculations to determine the most effective approach for practical clinical application. The contours generated by two distinct methods of atlas and neural network were compared to ground truth contours approved by a radiation oncologist, utilizing 13 distinct metrics. Furthermore, an innovative QA tool was conceptualized, designed for forthcoming cases based on the baseline dataset of 100 patient cases. The calculated metrics indicated that, in the majority of cases (60.58%), the neural network method demonstrated a notably higher alignment with the ground truth. Instances where no difference was observed accounted for 31.25%, while utilization of the atlas method represented 8.17%. The QA tool results showed that the two approaches achieved 100% agreement in 39.4% of instances for the atlas method and in 50.6% of instances for the neural network auto-segmentation. The results indicate that the neural network approach showcases superior performance, and its significantly closer physical alignment to ground truth contours in the majority of cases. The metrics derived from overlap and distance measurements have enabled clinicians to discern the optimal choice for practical clinical application.

## Introduction

Pediatric patients with central nervous system malignancies, such as medulloblastoma, ependymoma, atypical teratoid rhabdoid tumor, and germinoma, are often treated with craniospinal irradiation (CSI) therapy^1^. The most common malignant pediatric brain tumor is medulloblastoma, accounting for up to 25% of pediatric brain tumors diagnosed in high-income countries and up to 49% of pediatric brain tumors diagnosed in low and middle-income countries (LMICs). Additionally, medulloblastoma requires CSI treatment for patients older than 3–5 years of age^2^. Since CSI treats the entire brain and spinal volumes, treatment planning involves complex treatment fields and optimizations, where the dose to a significant number of normal structures must be considered to determine the risk of side effects. Treatment planning requires contouring these structures and tumor target volumes on simulation computed tomography (CT) scans. Delineating accurate structures is a time-consuming process critical for conformal therapies^3^. Due to the substantial number of necessary organ contours, CSI cases require significant time to contour and plan, which can be demanding, especially in LMICs where the radiation therapy capabilities can vary due to limited personnel or resources^3^. The necessity of CSI therapy to treat pediatric brain tumors leads to a need for fast and robust contouring methods.

Due to the time required for organ delineation and importance of accurate contours, auto-segmentation methods have been developed to decrease contouring time and reduce inter-observer variations^3^. Atlas techniques have emerged as a means to identify the most suitable reference case for a new patient from a repository of historical cases. Subsequently, these atlas methods utilize deformable image registration algorithms to adaptively transform the anatomical structure of the selected candidate to align with the new patient anatomy^4^. Atlas methods save manual segmentation time and increase consistency for differing cancers and treatment regions^3–5^. However, deformable image registration of patients from an atlas to a new patient can require minor or major editing to achieve clinically acceptable contours^5^ and have difficulties segmenting cases with anatomical variations or low contrast organs^6^. As the use of adaptive planning continues to expand, creating new contours between treatment fractions to account for anatomical changes further increases contouring time. In efforts to decrease the time required and increase the accuracy for segmentation in complex cases, artificial intelligence (AI) and machine learning auto-segmentation algorithms have been developed^6^. Deep learning algorithms have been shown to have higher delineation accuracy and lower execution time, including less manual correction time, than atlas auto-segmentation methods for organs at risk^7–10^. Deep learning models often create contours shown to be quantitatively more similar to ground truth contours by having lower distance metrics and higher geometrical similarity metrics, such as the 95% Hausdorff Distance (HD_95%_) and Dice Similarity Coefficient (DSC), respectively, as compared with atlas contours^7,10,11^. Although usually more accurate than atlas contours, deep learning contours of small organs or soft-tissue organs that have less contrast on a CT such as the optic chiasm, cochlea, and optic nerves are relatively poor as compared to ground truth contours^7,10^.

Deep learning algorithms must still be reviewed and corrected as necessary, which may take time, therefore automated ways to detect segmentation errors are desired to improve clinical use. Tools have been developed to detect these errors including comparing volumetric features, size, and shape^12,13^, a decision tree model based on image texture features^14^, and a group-wise inference system and anomaly detection modules scoring contours on shape, relational, and intensity features^15^. However, patients can exhibit a large variation in size, shape, and location of organs, which can lead to low accuracy for methods comparing these features^16^. The variability of patients is especially important when considering pediatric patients, since the wide range of ages treated leads to significant differences in size and shape of organs needing to be contoured. Deep learning neural networks have also been proposed to perform automated quality assurance of contouring which could improve clinical efficiency^17–19^. Hounsfield Units (HU) serve as a measurement scale within CT images, correlating pixel values in the image with tissue densities. Consequently, exploring the texture of tissue densities within a contour could serve as a safety measure, ensuring that unintended tissues, distinct from the intended feature of interest, are not inadvertently encompassed by an automated contouring method.

Currently, commercial auto-segmentation solutions tailored specifically for pediatric populations are unavailable. This absence is primarily attributed to the challenges posed by the scarcity of data and the variability in organ sizes within this demographic. These factors complicate the training of AI-based algorithms, which necessitate both a high volume and consistency in data to function effectively. Additionally, the validation of these algorithms poses its own set of challenges, as ensuring their accuracy and reliability across the varied anatomy of pediatric patients requires extensive and rigorous testing. To our knowledge, existing quality assurance (QA) tools for auto-segmentation primarily provide qualitative assessments or visual inspections of segmentation outcomes. This approach is due to the absence of ground truth contours, a limitation that is understandable. For a quantitative evaluation, ground truth contours are essential, yet these are not readily available. Consequently, we introduced an HU-based QA tool designed to address the lack of ground truth contours by leveraging historical data of ground truth contours as a knowledge-based QA tool.

The objective of this study is two-fold: to compare auto-segmentation tools employing an atlas approach and a deep learning method in the context of pediatric patients undergoing CSI treatment, and to introduce an innovative tool that leverages HU distributions for the purpose of QA in auto-segmentation algorithms. To our knowledge, commercial auto-segmentation models have not specifically been tested for pediatric CSI cases. Furthermore, no studies have been undertaken to capture the ground truth HU distributions of organs from previous cases and subsequently compare them with those of forthcoming pediatric CSI cases, serving as a knowledge-based QA tool.

## Results

### Contour comparison by organ

**Figure 1:**
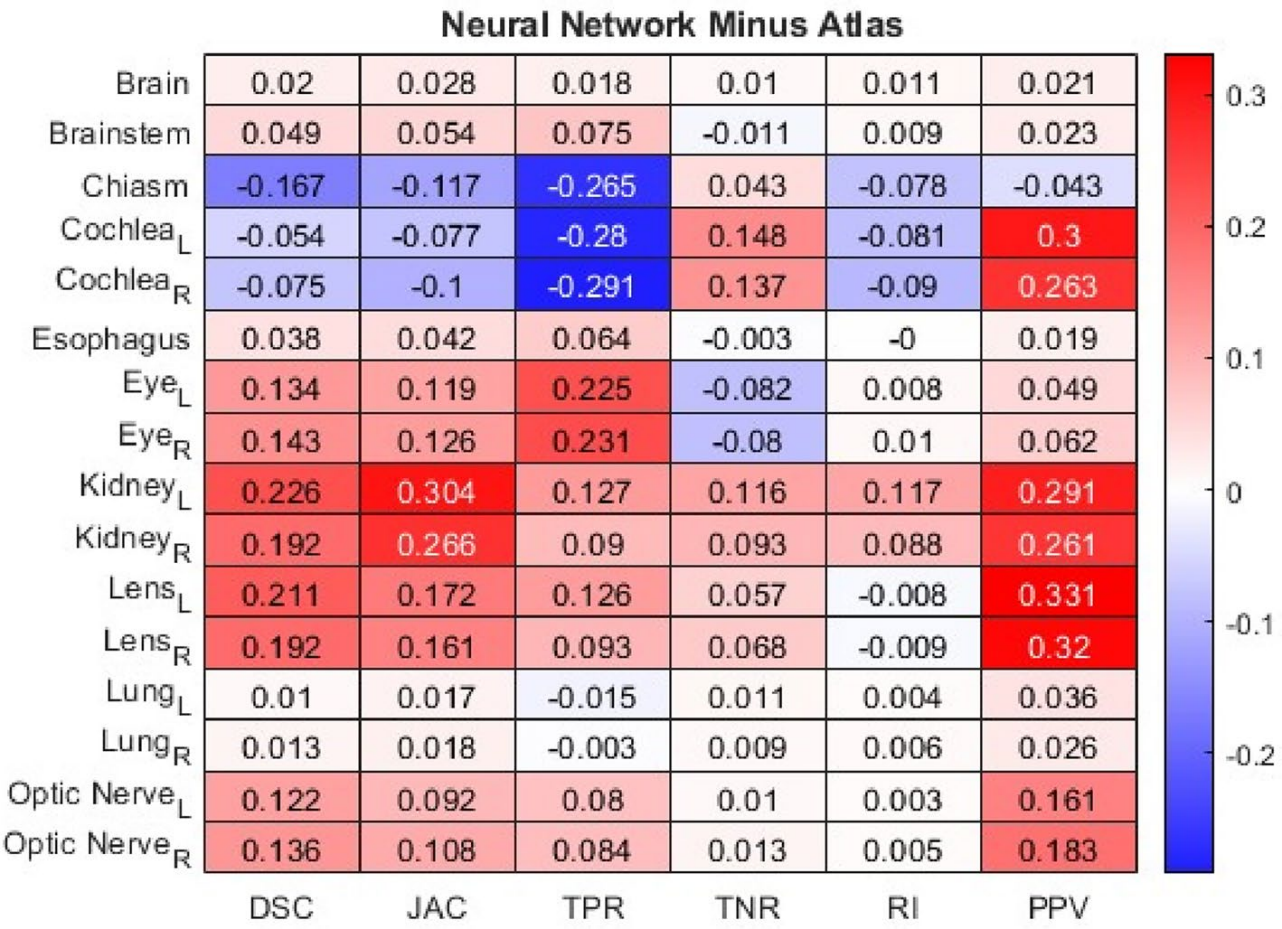
Heatmap showing the difference between neural network and atlas overlap metrics to determine if the atlas or neural network is closer to ground truth, designated by blue and red, respectively. L and R subscripts mean left and right, respectively. *DSC* dice similarity coefficient, *JAC* Jaccard index, *TPR* true positive rate, *TNR* true negative rate, *RI* rand index, *PPV* positive predictive value.

Each of the 13 metrics was computed for all 16 organ contours across a sample of 100 patients. The age of these patients spanned from 2 to 25 years, with a median age of 8, encompassing a diverse range of ages and patient sizes. This age spectrum was chosen to ensure that the study comprehensively evaluated the performance of the auto-segmentation tools across a broad demographic. To compare the outcomes of atlas and neural network auto-segmentation, the calculated metrics were averaged across the entire cohort of 100 patients. This analysis aimed to examine the performance of each method across various organs. For a visual representation of the comparison between the two approaches, heatmaps were generated. Figure 1 showcased a heatmap that portrayed the difference between neural network and atlas overlap metrics. Given that overlap metrics fall within the 0 to 1 range, where 1 indicates the closest alignment with ground truth, a positive value indicated that the neural network method was more aligned with ground truth, whereas a negative value indicated that the atlas method was more aligned with ground truth. The color bar displayed darker hues for cases where the methods were highly dissimilar, with red signifying the neural network method's closer alignment to ground truth and blue indicating the atlas method's closer alignment to ground truth. Lighter hues, on the other hand, represented greater similarity between the methods.

**Figure 2:**
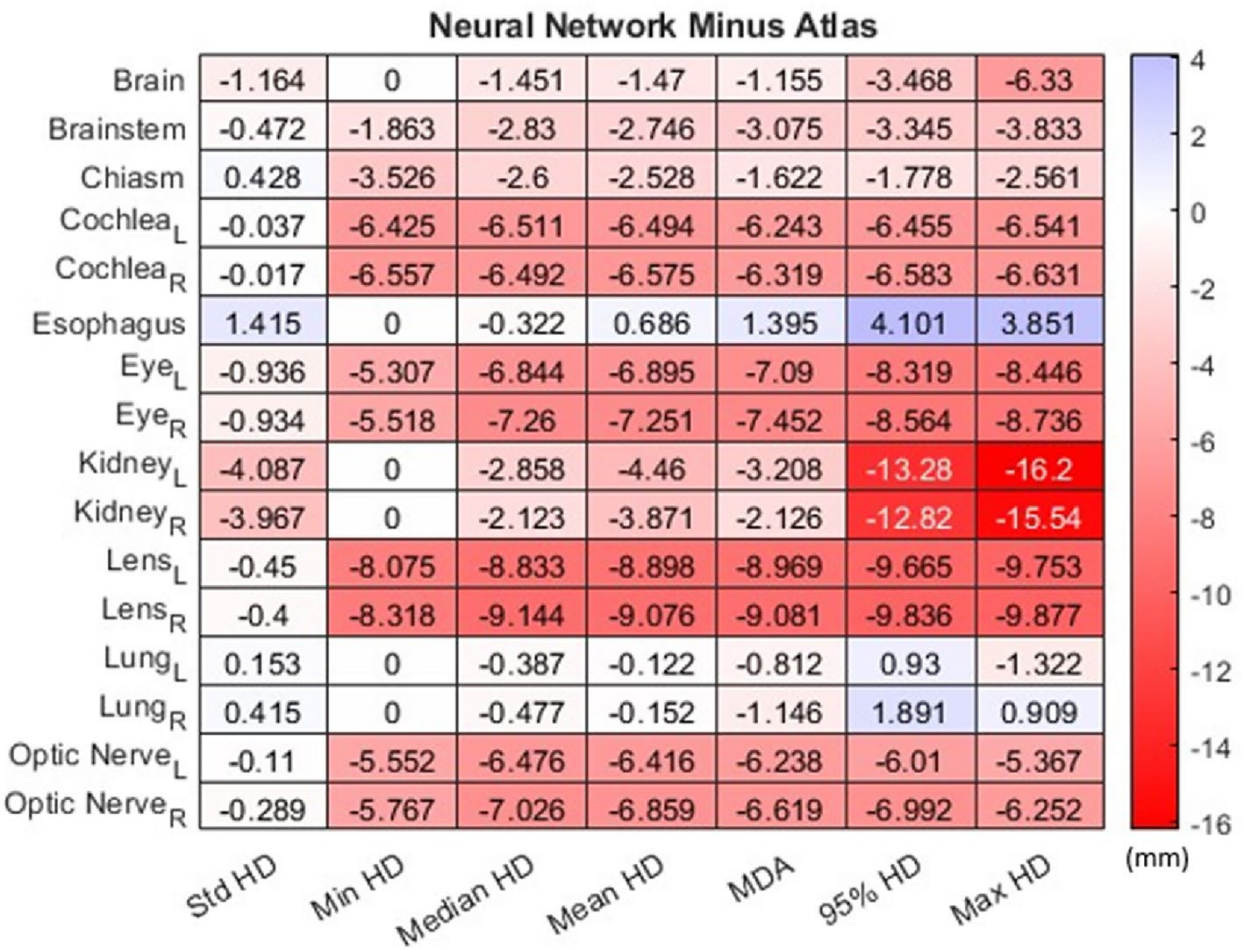
Heatmap showing the difference between neural network and atlas distance metrics to determine if the atlas or neural network is closer to ground truth, designated by blue and red, respectively. L and R subscripts mean left and right, respectively. *HD* Hausdorff distance, *MDA* mean distance to agreement.

A similar heatmap illustrating the distinction between neural network distance metrics and atlas distance metrics, was featured in Fig. 2. Considering that distance metrics approach ground truth as the distance approaches 0 mm, a negative value indicated that the neural network method was more closely aligned with ground truth, while a positive value denoted that the atlas method was more closely aligned with ground truth. The color bar visualization indicated that darker hues corresponded to greater dissimilarity between the methods, with red signifying the neural network method's closer alignment to ground truth and blue indicating the atlas method's closer alignment to ground truth. Conversely, lighter hues indicated increased similarity between the methods.

The instances in which the atlas method displayed accuracy in generating contours closer to ground truth were limited to the chiasm and cochleae, except for the TNR metric. For the remaining organs, the neural network method either matched the performance of the atlas method or exhibited higher accuracy in creating contours that were closer to ground truth. Overall, when comparing the two methods, the neural network demonstrated closer alignment with ground truth in 60.58% of cases, the atlas method was superior in 8.17% of cases, and no significant difference was observed in 31.25% of cases.

### Contour comparison by patient

**Figure 3:**
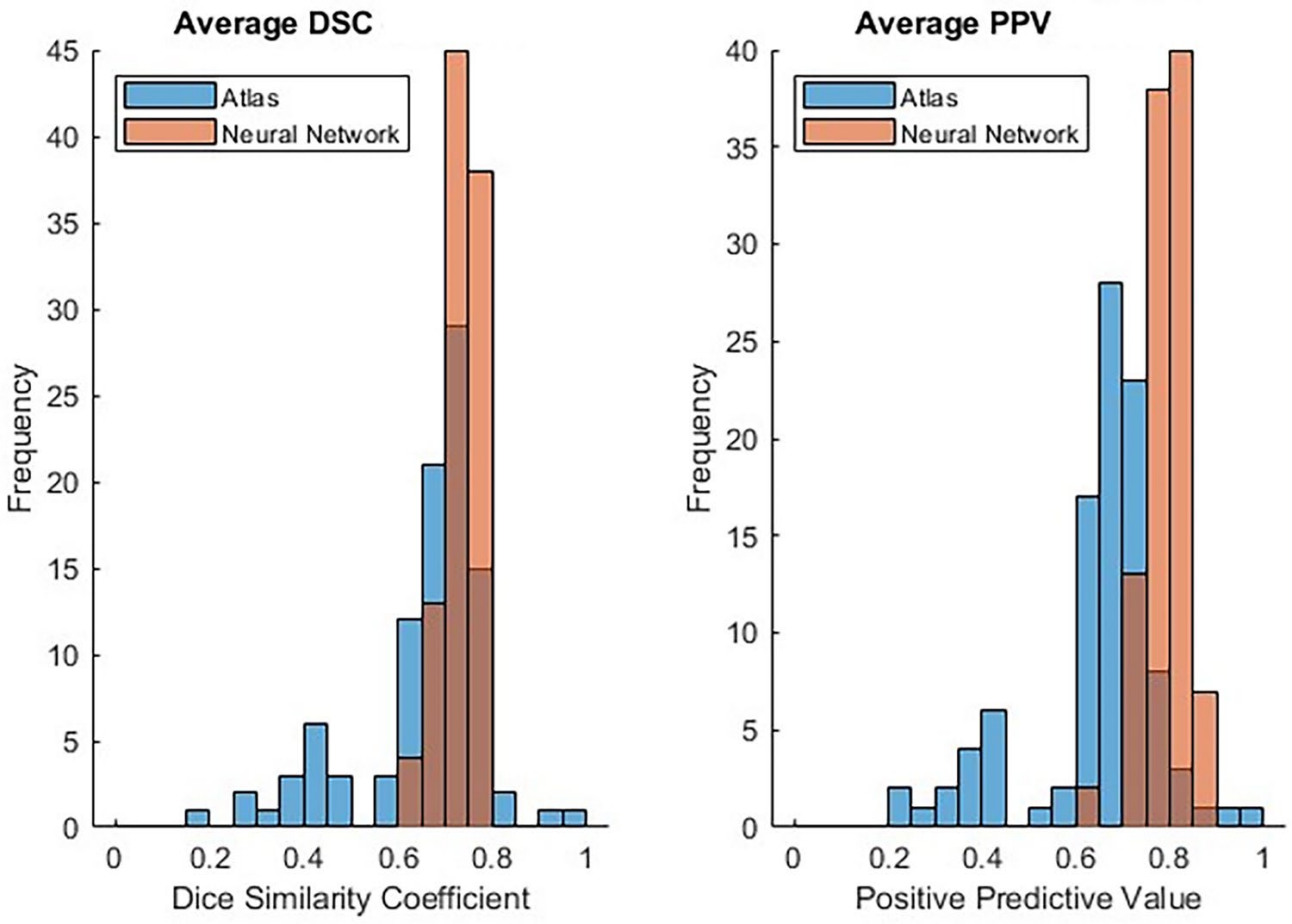
Histograms showing the dice similarity coefficient (DSC) and positive predictive value (PPV) for atlas and neural network methods in 100 patients averaged across all 16 organs.

To conduct a more comprehensive comparison between atlas and neural network auto-segmentation outcomes, the metrics were averaged across organs, yielding an average metric for each patient. This approach enables an analysis of the methods' performance in contouring an entire patient. The collective metrics encompassing the entire body are outlined in Table 1. Specifically, the average DSC for all organs was 0.66 ± 0.14 for the atlas method and 0.73 ± 0.04 for the neural network method. Correspondingly, the average PPV for all organs was 0.65 ± 0.14 for the atlas method and 0.79 ± 0.04 for the neural network method. A comparison of the DSC and PPV distributions between the two methods was presented in Fig. 3. The average mean HD for all organs was found to be 6.16 ± 17.21 mm for the atlas method and 1.59 ± 1.08 mm for the neural network method.

**Table 1:** 13 quantitative metrics averaged across 16 organs for all 100 patients and compared against ground truth contours for atlas and neural network methods.

Metrics	Atlas	Neural network
DSC	0.6585 ± 0.1402	0.7329 ± 0.0360
JAC	0.5521 ± 0.1247	0.6279 ± 0.0412
TPR	0.6998 ± 0.1524	0.7221 ± 0.0439
TNR	0.9010 ± 0.0293	0.9348 ± 0.0186
RI	0.8746 ± 0.0246	0.8743 ± 0.0165
PPV	0.6490 ± 0.1401	0.7931 ± 0.0431
Std HD [mm]	2.2873 ± 1.6448	1.6341 ± 0.5598
Min HD [mm]	3.7211 ± 15.2225	0.1644 ± 0.9687
Median HD [mm]	5.6303 ± 16.8567	1.1533 ± 1.0256
Mean HD [mm]	6.1605 ± 17.2076	1.5900 ± 1.0787
MDA [mm]	5.9474 ± 17.1572	1.5874 ± 1.1603
95% HD [mm]	10.6224 ± 20.0135	4.9856 ± 2.0027
Max HD [mm]	15.6797 ± 20.5514	9.2653 ± 2.3628

*DSC* dice similarity coefficient, *JAC* jaccard index, *TPR* true positive rate, *HD* Hausdorff distance, *MDA* mean distance to agreement, *TNR* true negative rate, *RI* rand index, *PPV* positive predictive value.

For each combination of organ and metric (e.g. Brain + DSC), a paired t-test was conducted to compare the atlas method with the neural network method. The null hypothesis maintained that there was no significant difference between the two methods, setting a significance threshold at 0.05. The analysis of p-values indicated that, in 208 comparisons, the neural network method outperformed the atlas method in 137 instances, the atlas method prevailed in 17 instances, and in 54 instances, there was no significant difference between the performances of the neural network and atlas methods, adhering to the established significance level of 0.05.

### Knowledge-based quality assurance tool

**Figure 4:**
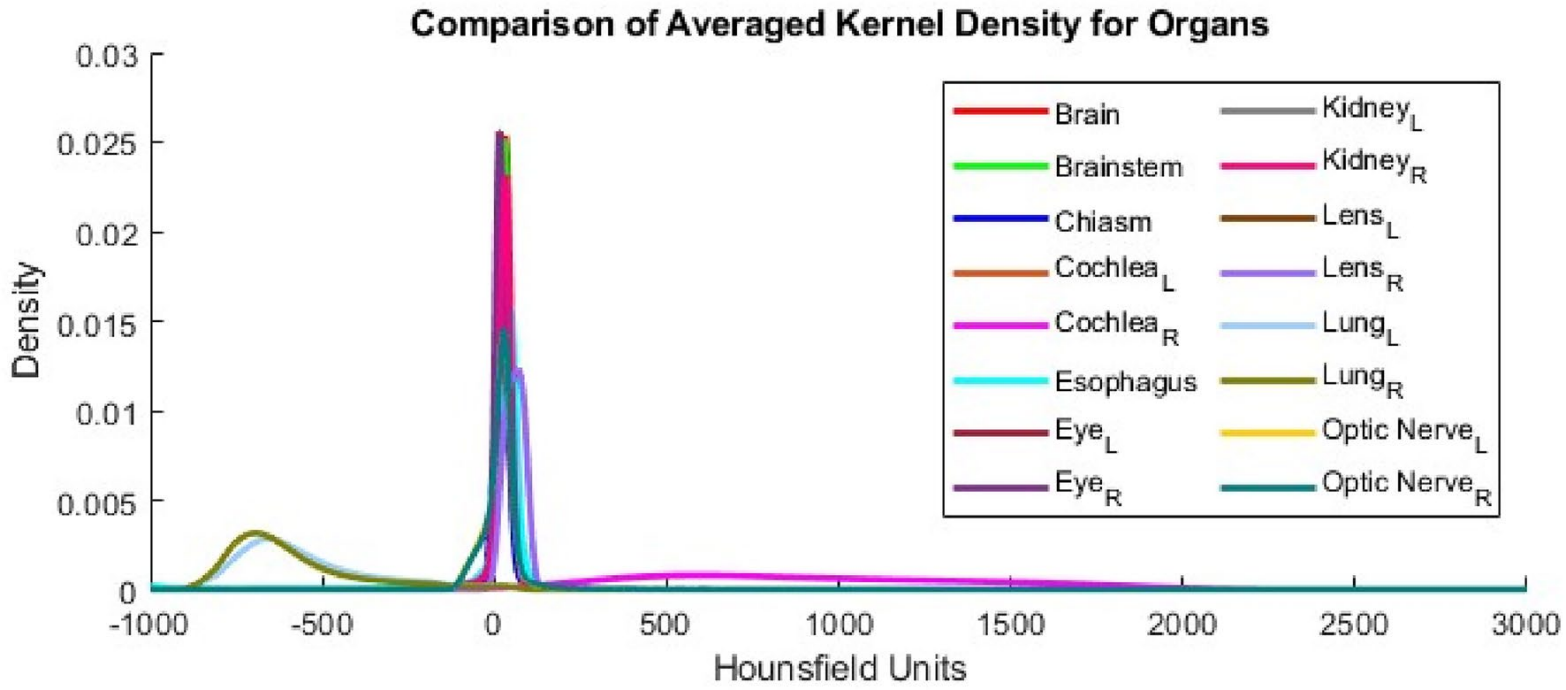
Average baseline KDE distributions for all 16 organs of 100 CSI patients. L and R subscripts mean left and right, respectively.

**Figure 5:**
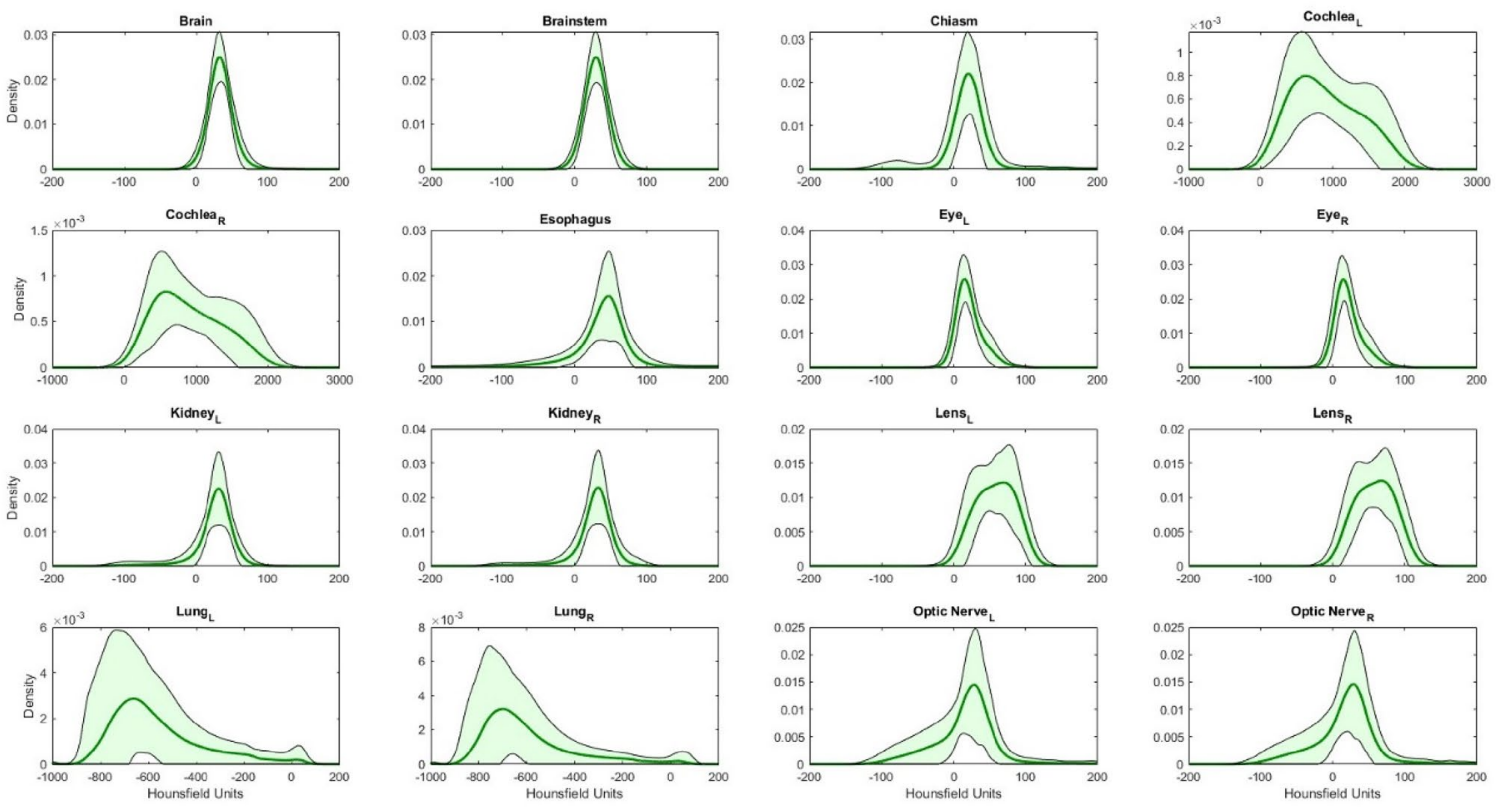
Baseline KDE distributions with ± 2 SD (green shaded area) for 16 organs of 100 CSI patients. L and R subscripts mean left and right, respectively.

The baseline KDEs for each organ are visualized for 100 CSI patients in Fig. 4. The HU distributions were distinct, each varying over a specific range of HU values. The standard KDEs included two standard deviations above and below the average KDE to include 95% of ground truth contours. These KDEs with the standard deviation bounds are shown in Fig. 5.

**Figure 6:**
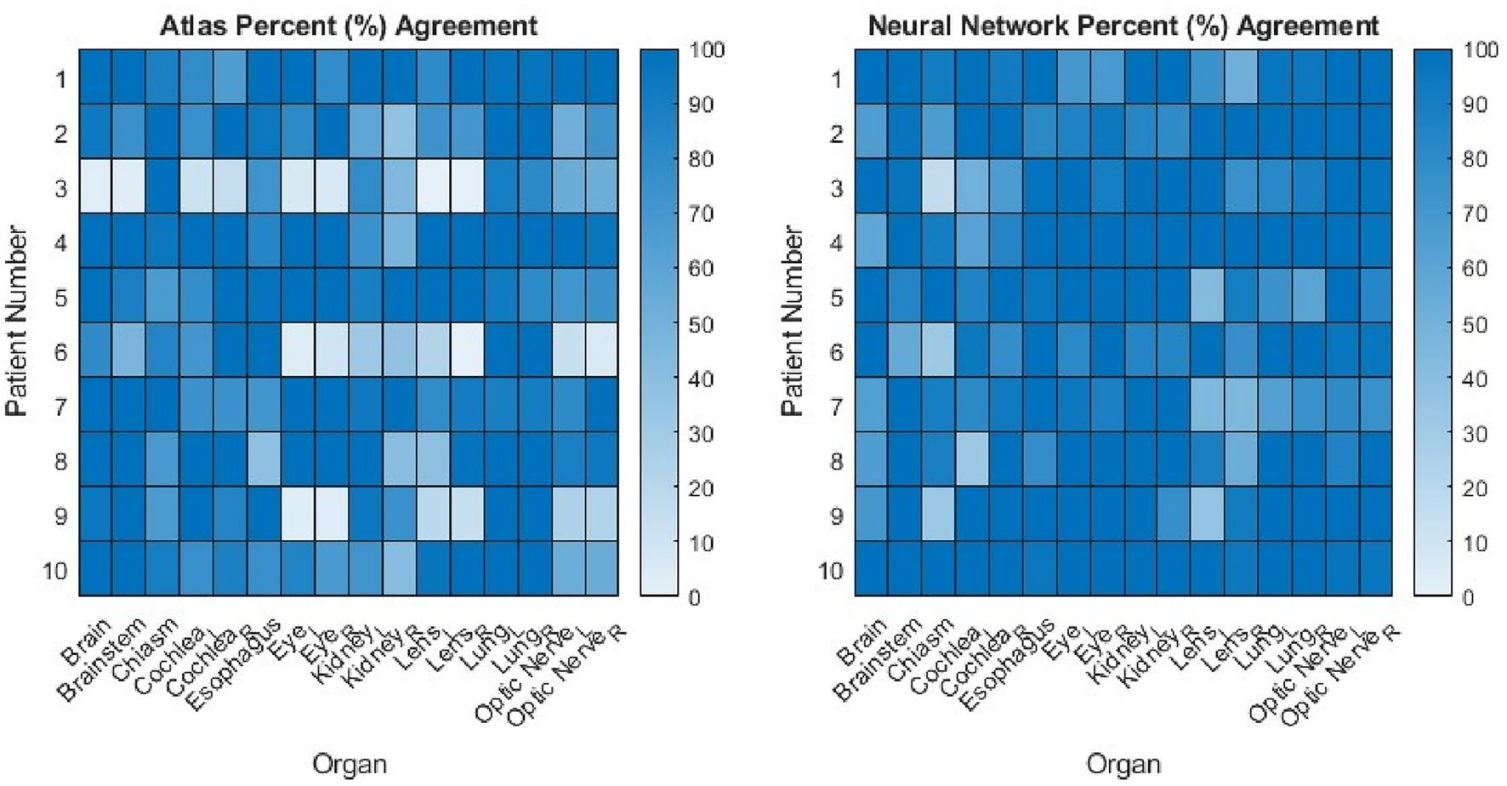
Percentage agreements of atlas and neural network methods against baseline KDEs for 10 test patients and 16 organs. L and R subscripts mean left and right, respectively.

In total, 160 auto-segmentations were performed for both atlas and neural network methods for 10 test patients and 16 organs and compared against KDE baselines from 100 patients. The results showed that the two approaches achieved agreements of over 75% in 65.6% of instances for the atlas method and in 82.5% of instances for the neural network auto-segmentation. Moreover, the atlas method achieved agreements for 100% in 39.4% of cases, while the neural network method achieved 100% agreement in 50.6% of cases. Percent agreements were presented in heat maps for two methods in 10 test patients and 16 normal organs in Fig. 6.

## Discussion

### Contour comparison by organ

The neural network method produced contours that were generally closer to the ground truth, except for certain cases like the chiasm and cochleae. This observation aligns with existing literature^9^, which indicates that neural networks tend to struggle with small structures and those with low contrast—attributes that characterize the cochleae and chiasm, respectively. Notably, manual contouring is often required for the chiasm in CSI patients due to its absence in CT scans and its visualization primarily in MRI scans. Moreover, the neural network's chiasm contours often took on a rectangular form, in contrast to the more accurate "X" shape of the ground truth contours, potentially contributing to discrepancies. For the chiasm and cochleae, the atlas method tended to overestimate size, resulting in a high TPR as the model captured a significant portion of the actual feature voxels. Conversely, the neural network's contours leaned toward underestimation, leading to lower TPR and other metrics, yet a higher TNR due to fewer false positives. Furthermore, the neural network's cochlea contours exhibited notably better PPV, implying that the model's predictions largely aligned with actual cochlear components. Variations in contouring approaches among radiation oncologists might contribute to these findings. Comparatively, the atlas method yielded higher TNR for the brainstem, esophagus, and eyes. This might arise from the neural network's lack of training on the pediatric population, potentially resulting in size overestimation. This is indicated by higher TPR and PPV for the neural network, signifying accurate prediction of voxels relevant to the target structure.

The paired t-test, conducted at a significance level of 0.05, between the atlas and neural network methods, demonstrated that the neural network significantly outperformed the atlas method in achieving contour accuracy for kidneys and lenses, as supported by most metrics. Among the evaluated 16 organs, the neural network showed statistically significant enhancements in DSC for 9 organs (56.25%) in comparison with the atlas method. For 4 organs (25%), the DSC results were similar between the two methods. On the whole, the neural network presented contours that were either more aligned with the ground truth or showed fewer discrepancies across all metrics assessed, relative to the atlas method.

### Contour comparison by patient

Patient-wise average metrics for organs exhibited higher values in terms of overlap metrics (excluding RI) and lower values for distance metrics with the neural network method compared to the atlas method, signifying that the neural network's results were consistently closer to the ground truth across almost all metrics. Instances where the atlas method outperformed feature prediction occurred in fewer than 3% of cases, potentially arising from high patient matching facilitating successful and precise deformable registration. However, predominantly, the atlas method's PPV values were considerably lower, particularly at lower percentiles. While the neural network might not completely anticipate contours with high similarity to ground truth for all patients, its predictions pertaining to organs or structures are highly likely to be accurate due to its elevated PPV. Consequently, this may lead to fewer inadvertent errors and necessitate less editing time compared to the atlas method. Even though the neural network method wasn’t specifically trained on pediatric cases, it still benefits from the entirety of training patient data. Conversely, the atlas method constructs structures solely based on a single prior patient due to one-on-one deformable image registration method between the best-matched anatomy and the test case. As a result, the neural network method is likely more informed, adaptive, and capable of contour creation, owing to its comprehensive training data. Developing a similar model trained on pediatric CSI patients could potentially yield even more comparable contours to the ground truth.

### Knowledge-based quality assurance tool

**Figure 7:**
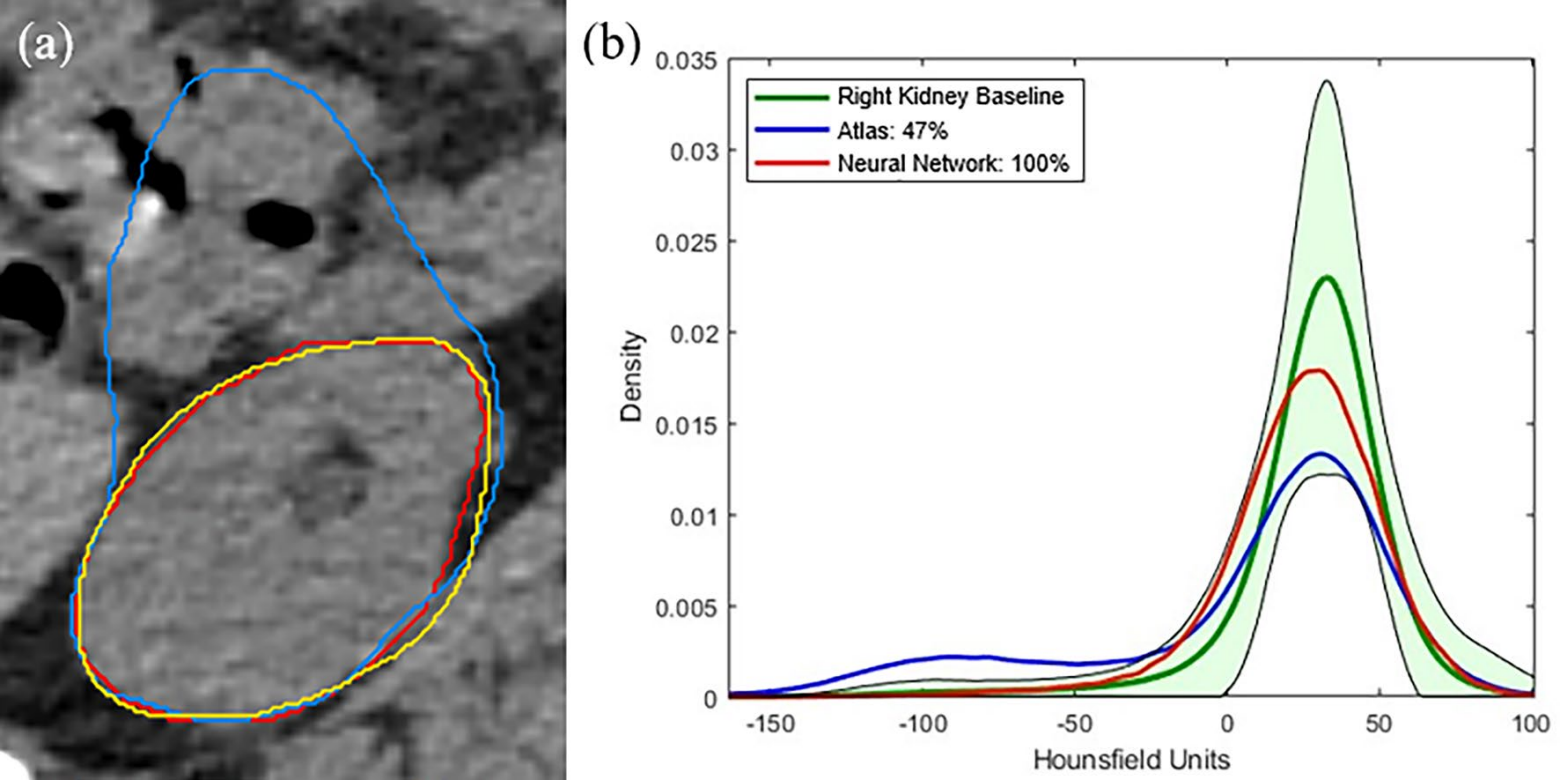
Single axial CT slice shows the overlaps of yellow line (ground truth), blue line (atlas), and red line (neural network) methods for right kidney in (**a**). KDEs show percent agreements between the methods against the baseline KDE for right kidney for patient #4 in (**b**).

One application of knowledge-based quality assurance was demonstrated on a test patient (patient #4) concerning the right kidney, out of the total of 10 patients in Fig. 7. The comparison of contours between the atlas and neural network auto-segmentation techniques against the reference contour is depicted in Fig. 7a. The objective was to ascertain the percentage agreement between the two methods, leveraging the baseline KDEs derived from 100 CSI patients, as illustrated in Fig. 7b. The discernible disparity in the right kidney's contour resulting from the atlas method was highly pronounced and quantitatively characterized using the KDE approach, which involved modeling the HU distribution values from the past 100 CSI cases.

The applicability of the in-house knowledge-based QA tool remains independent of the specific type of auto-segmentation method employed. However, a key constraint lies in the fact that the accuracy of KDEs is contingent upon the subjectivity inherent in the ground truth contours endorsed by clinicians. The principal aim of utilizing this tool is to identify significant errors stemming from auto-segmentation processes. A clinical threshold of 100% agreement could be pursued. Organs falling below this threshold are highlighted for closer scrutiny and potential further investigation. This threshold serves as an indicator for the presence of gross errors generated by the auto-segmentation tools. This assessment could be seamlessly incorporated into the review stage of treatment planning, ensuring that any organ segmentations falling below the threshold are promptly flagged for clinician review. The adoption of this tool in clinical practice may encounter several barriers. One significant barrier is the resistance to change, common in settings where traditional practices are deeply ingrained. To mitigate this, extensive training, and demonstrations of the tool's efficacy in enhancing patient safety and improving workflow efficiency could be conducted. Additionally, integration challenges with existing image manipulation systems may arise, necessitating collaboration with IT professionals to ensure compatibility and ease of use. Further testing is warranted to comprehensively gauge the tool's applicability and ascertain the optimal approach for leveraging it as a safety measure for auto-segmentation models. Our findings indicate that 10 test cases fell within the 2-sigma region when compared to 100 historic cases. This suggests that the distribution of results could vary across different centers, potentially being tighter or more relaxed depending on the consistency with which ground truth contours are executed. To accommodate this variability, the tolerance level of 100% in the QA tool can be adjusted based on several factors, including the number of normal organs reviewed, the percentage of gross errors identified, and the clinically acceptable deviations from the expected results.

## Conclusion

Neural networks hold great promise for automating the segmentation process in radiation oncology. This automation is particularly valuable in complex treatment planning scenarios like CSI, where the process is time-consuming and resource-intensive. This quantitative evaluation, comparing a neural network approach to an atlas-based method, revealed that, on the whole, the neural network generated contours that were closer to the ground truth. Notably, the neural network model assessed in this study, trained on adult patients, exhibited respectable performance on pediatric cases as well. However, even greater advancements could potentially arise from employing a neural network specifically trained on pediatric patients.

In the context of safety and quality assurance, a knowledge-based comparison of HU distributions offers a valuable means to scrutinize auto-segmentation outcomes. This approach involves verifying if organ contours align with established clinical standards, effectively identifying any inadvertent inclusion of surrounding tissues within the contour. The integration of a neural network-based auto-segmentation algorithm with such a quality assurance tool has the potential to significantly enhance the efficiency of contouring workflows, not only for CSI patients but across a broader spectrum of cases.

## Methods

### Patient data selection

This study was performed by using retrospectively acquired CT scans on Phillips IQon Spectral CT (Philips Healthcare, Cleveland, OH) and commercially available clinical MIM software (MIM, version 7.3.2, MIM Software Inc., Cleveland, OH). The datasets of pediatric patients who underwent CSI treatments were randomly selected from November, 2015 to August, 2023. Institutional review board approval was obtained prior to the study. Patient ages were from 2 to 25 years, with a median age of 8. The patients had CT acquisition using 120 kVp, 1.171 spiral pitch factor, 0.625 mm collimation, a 50 cm field of view, 512 × 512 matrix size, and slice thickness of 1.5 mm based on CSI protocol.

For each patient, dosimetrists first manually delineated the normal structures with great care, followed by a thorough review of their work. After this initial phase, physicians made any necessary adjustments and provided their final approvals. This step-by-step collaboration between dosimetrists and physicians guaranteed that the contours of normal organ structures met the high clinical standards required for treatment planning. This process established the ground truth contours for 170 patients including brain, brainstem, chiasm, left cochlea, right cochlea, esophagus, left eye, right eye, left lens, right lens, left lung, right lung, left kidney, right kidney, left optic nerve, and right optic nerve.

Automated segmentation was applied to the CT scans of the CSI patients through an atlas segmentation software, employing a deformable image registration algorithm known as the VoxAlign Deformation Engine, developed by MIM Software. The atlas algorithm searched an archive containing 170 prior CSI ground truth cases and identified the CT scan that closely matched the current patient's anatomy. By minimizing intensity disparities between the two images through deformable image registration of the historical patient's CT, the algorithm generated a deformed vector field. This vector field was then employed to modify the contours from the previous patient, aligning them with the anatomical specifics of the current patient^20^. The atlas algorithm was applied to 100 CSI test cases to establish the atlas test contours.

The CT scans of these 100 CSI test patients were also auto-segmented using a neural network algorithm (Contour ProtégéAI, MIM Software Inc., Cleveland, OH) to establish the neural network test contours. For each patient, the neural network algorithm was executed, employing a U-Net architecture to convert the input image into a segmentation mask for each specific anatomical structure^21^. Both the CT Head and Neck neural network and CT Thorax models (Contour ProtégéAI, MIM Software Inc., Cleveland, OH) were used for each patient. These individual contours were aggregated, resulting in a collection of 16 listed contours for 100 test patients that were recorded for the purpose of comparison.

### Contour comparison

100 CSI patients that were auto-segmented by both atlas and the neural network methods were analyzed using 13 distinct metrics and compared against the ground truth contours. The metrics can be split into two categories, overlap and distance measures. The crisp definitions for all metrics were adapted from a study^22^.

### Overlap metrics

The segmentations of the ground truth and the test were defined as ${S}_{g}$ and ${S}_{t}$, respectively. Both segmentations were split up into two classes, ${S}_{g}= \left\{{S}_{g}^{1},{S}_{g}^{2}\right\}$ and ${S}_{t}= \left\{{S}_{t}^{1},{S}_{t}^{2}\right\}$, where the first class $\left({S}_{g}^{1},{S}_{t}^{1}\right)$ was the anatomy of interest and the second class $\left({S}_{g}^{2},{S}_{t}^{2}\right)$ was the background. An assignment function ${f}_{g}^{i}(x)$ defined if a point $x$ in a medical image volume was in the feature of interest of the background, where ${f}_{g}^{i}\left(x\right)=1$ if $x \in {S}_{g}^{i}$, and ${f}_{g}^{i}\left(x\right)=0$ if $x \notin {S}_{g}^{i}.$ The definition of ${f}_{t}^{i}(x)$, the assignment function for the test segmentation, was defined similarly. If $X=\left\{{x}_{1},\dots ,{x}_{n}\right\}$ defined the point set of all points inside of the medical image volume, all points in the image were members of the feature of image or background classes, meaning ${f}_{g}^{1}\left(x\right)+{ f}_{g}^{2}\left(x\right)=1$ and ${f}_{t}^{1}\left(x\right)+{ f}_{t}^{2}\left(x\right)=1$ for all $x \in X$.

The common cardinalities describing the overlap of the two segmentations were the four aspects of a confusion matrix, including true positives (*TP*), false positives (*FP*), true negatives (*TN*), and false negatives (*FN*). These were defined by the sum of agreement between the classes of segmentation of $i\in {S}_{g}$ and $j\in {S}_{t}$, calculated as

$${m}_{ij}=\sum_{r=1}^{\left|X\right|}{f}_{g}^{i}\left({x}_{r}\right){f}_{t}^{j}\left({x}_{r}\right),$$

where $$TP= {m}_{11}, FP={m}_{10}, FN= {m}_{01}$$, and $$TN= {m}_{00}$$.

True positives described the instances that were part of the feature of interest and were correctly classified as part of the feature of interest by the test segmentation. False positives described the instances that were part of the background and were incorrectly classified as part of the feature of interest by the test segmentation. False negatives described the instances that were part of the feature of interest and were incorrectly classified as background by the test segmentation. True negatives were describing the instances that were part of the background and were correctly classified as background by the test segmentation.

The overlap measures used these cardinalities. All overlap measures ranged from 0 to 1, where 0 indicated low agreement between ground truth and test segmentations and 1 indicated high agreement between ground truth and test segmentations. The Dice Similarity Coefficient (DSC), commonly used in comparing medical volume segmentation, measured the degree of overlap between two segmentations. It is defined by

$$DSC=\frac{2\times \left|{S}_{g}^{1} \cap {S}_{t}^{1}\right|}{\left|{S}_{g}^{1}\right| + \left|{S}_{t}^{1}\right|}=\frac{2TP}{2TP+FP+FN}.$$

The Jaccard index (JAC) measured overlap and can be related to DSC by,

$$JAC=\frac{\left|{S}_{g}^{1} \cap {S}_{t}^{1}\right|}{\left|{S}_{g}^{1} \cup {S}_{t}^{1}\right|}=\frac{TP}{TP+FP+FN}=\frac{DSC}{2-DSC}.$$

The true positive rate (TPR), also known as Sensitivity or Recall, measured the proportion of positive cases correctly identified by the test segmentation and is defined by,

$$Recall = Sensitivity = TPR =\frac{TP}{TP+FN}.$$

The true negative rate (TNR), also known as Specificity, measured the proportion of negative cases correctly identified by the test segmentation and is defined by,

$$Specificity = TNR =\frac{TN}{TN+FP}.$$

The positive predictive value (PPV), also known as Precision, represented the proportion of predicted positive cases that were actually positive and is defined by,

$$Precision= PPV =\frac{TP}{TP+FP}.$$

The rand index (RI) is commonly used to measure similarity between data clustering but can also evaluate classifications. The RI described the proportion of correct identifications by the test segmentation and is defined by,

$$RI =\frac{TP+TN}{TP+TN+FP+FN}.$$

It was noted during the calculations that since the entire CT was used as an input for both the ground truth and test segmentations that the number of true negatives (TN) was extremely high, causing the TNR and RI to be very close to 1 (e.g. 0.99998) even if a segmentation had a low TPR and PPV (e.g. below 0.2). To allow for the TNR and RI to provide meaningful data, the segmentation comparisons were performed after a bounding box was created. The bounding box was the smallest rectangular volume enclosing both ground truth and test segmentations. Note that this bounding box did not have any effect on TP, FP, or FN since all actual positives and predicted positives were included in the bounding box.

### Distance metrics

The next set of metrics were the distance metrics, which used the Hausdorff Distance (HD), a measure of the distance between the ground truth and test segmentations. The HD measured the maximum of the distances describing each point in both sets of surface points of each volume to its closest point in the other set of surface points and is defined by,

$$HD=\underset{\text{max}}{\left(h\left(A,B\right),h(B,A)\right) ,}$$

where $h\left(A,B\right)$ is the directed Hausdorff Distance between point set A and point set B described by,

$$h\left(A,B\right)=\underset{a\in A}{{\text{max}}}\underset{b\in B}{{\text{min}}}\Vert a-b\Vert ,$$

where $\Vert a-b\Vert$ is Euclidean distance between two points. However, the HD was sensitive to outliers, so other metrics were developed to compare the distance. The other distance metrics were calculated from the nearest neighbor distances for all points in A and all points in B. This nearest neighbor function, returning a vector of distances describing the minimum distances from set A to B, can be written as:

$$d\left(A,B\right)= \underset{b\in B}{{\text{min}}}\Vert a-b\Vert  {\text{for}}\,a\,{\text{in}}\,A.$$

The traditional HD was designated as ${HD}_{max}$ which can also be written as,

$${HD}_{max}={\text{max}}\left(d\left(A,B\right),d\left(B,A\right)\right),$$

where $d\left(A,B\right)$ and $d\left(B,A\right)$ are vectors. The other distance metrics were calculated as follows:

$${HD}_{std}={\text{std}}\left(d\left(A,B\right),d\left(B,A\right)\right),$$

$${HD}_{min}={\text{min}}\left(d\left(A,B\right),d\left(B,A\right)\right),$$

$${HD}_{mean}={\text{mean}}\left(d\left(A,B\right),d\left(B,A\right)\right),$$

$${HD}_{median}={\text{median}}\left(d\left(A,B\right),d\left(B,A\right)\right),$$

$${HD}_{95\%}={{\text{percentile}}}_{95\%}\left(d\left(A,B\right),d\left(B,A\right)\right).$$

The mean distance to agreement (MDA) measured the mean of the nearest neighbors from only one set of surface points to the other as described by

$$MDA={\text{mean}}\left(d\left(A,B\right)\right).$$

### Knowledge-based quality assurance tool

An in-house knowledge-based quality assurance tool was developed by leveraging the distinctive patterns of HU distributions for each individual organ. Sample HU distributions were acquired for each ground truth contour, a total of 1600 HU distributions encompassing 100 patients for 16 organs. The QA tool used kernel density estimation (KDE) which is a statistical technique to estimate the probability density function of a random variable and provide insights into the underlying distribution of data points. In a CT image, the data points were interpreted as voxels, each accompanied by a corresponding HU value. To generate a KDE for each patient's organs, a collection of HU values was extracted. This KDE approach utilized kernel densities to construct a probability density function, effectively capturing the normalized distribution so that the total area under the probability distribution is equal to 1. This methodology permitted a comparison that remained uninfluenced by the organs’ varying sizes, which can differ widely within a pediatric population.

To validate the consistency of the HU distributions across multiple patients, KDEs were randomly divided into two groups. One group consisted of KDEs for 80 patients, which were utilized to establish a reference baseline distribution. The second group comprised KDEs for 20 patients, intended for validation purposes.

Subsequently, KDSs for the cohort of 80 reference patients were collectively calculated for each organ. This procedure yielded 16 averaged KDEs, corresponding to the 16 distinct organs. The standard deviation (SD) among these KDEs across the reference patient group was also determined. The agreement value of 0.95 was observed when the ground truths of 20 test patients were compared against the reference KDEs of 80 patients. This correspondence directly conformed to the statistical empirical guideline referred to as the two SDs or two-sigma rule. Therefore, it was logical to anticipate that the newly generated contours would fall within the range of ± 95% of the ground truth HU distributions.

To test the agreements of the newly auto-segmented contours, KDEs were created for two datasets: the first one was comprised of KDEs from 100 patients, utilized to establish a baseline distribution of ground truth contours. The second dataset consisted of KDEs from 10 patients who were not included in the library of auto-segmentation tools. These 10 patients’ data were segmented by both atlas and neural network methods for further analysis. The KDEs from 100 patients for each organ were averaged, resulting in the creation of 16 benchmark KDEs corresponding to the 16 organs. These averaged KDEs were employed as the baseline distribution for each respective organ. Upper bounds for the baselines were determined by adding two SDs to the distributions, while lower bounds were established by subtracting two SD from the distributions. These upper and lower bounds encompassed 95% of the ground truth distributions at each HU value, adhering to the empirical 2-sigma rule.

Test contours from atlas and neural network methods were compared against the baseline distribution using ± 2 SD values as part of the knowledge-based quality assurance procedure. This comparison was performed by creating a KDE for the test contour from its HU distribution. The extent of the test KDE, denoted as ${KDE}_{T}$, which falls within the lower and upper bounds of the baseline KDE, represented as ${LB}_{{KDE}_{B}}$ and ${UB}_{{KDE}_{B}}$ respectively, was calculated. This calculation yielded a quantitative measure that gauged the level of agreement, as formulated below.

$$Agreement \,\,value= \frac{{\sum }_{i}{LB}_{{KDE}_{B}}\le {KDE}_{T}(i)\le {UB}_{{KDE}_{B}}}{length({KDE}_{T})}.$$

The agreement values between the ground truth belonging to 100 cases and 10 test distributions were computed for each organ. This procedure was carried out to verify whether the agreement value with the baseline distribution effectively represented the quality of auto-segmented contour sets. Based on the agreement values, the discrepancies were investigated between ground truth and auto-segmented contour pairs.

### Institutional review board statement

The study was conducted in accordance with the Declaration of Helsinki and approved by the Institutional Review Board (IRB) (IRB number: 23-1456 and date of approval: 8/3/2023) of St. Jude Children’s Research Hospital, Memphis, TN.

### Informed consent

Informed consent was waived due to the nature of retrospective study by the Institutional Review Board (IRB) (IRB number: 23-1456 and date of approval: 8/3/2023) of St. Jude Children’s Research Hospital, Memphis, TN.

## Data Availability

The data that support the findings of this study are available from the corresponding author, upon reasonable request.

## References

[1] Turcas, A., Kelly, S. M., Clementel, E. & Cernea, D. Tomotherapy for cranio-spinal irradiation. *Clin. Transl. Radiat. Oncol.* **38**, 96–103. 10.1016/j.ctro.2022.11.003 (2022). 36407491 10.1016/j.ctro.2022.11.003 PMC9672131

[2] Hernandez, S. *et al.* Automating the treatment planning process for 3D-conformal pediatric craniospinal irradiation therapy. *Pediatr. Blood Cancer* **70**(3), e30164. 10.1002/pbc.30164 (2023). 36591994 10.1002/pbc.30164

[3] Daisne, J. F. & Blumhofer, A. Atlas-based automatic segmentation of head and neck organs at risk and nodal target volumes: A clinical validation. *Radiat. Oncol.* **8**, 154. 10.1186/1748-717X-8-154 (2013). 23803232 10.1186/1748-717X-8-154 PMC3722083

[4] Young, A. V., Wortham, A., Wernick, I., Evans, A. & Ennis, R. D. Atlas-based segmentation improves consistency and decreases time required for contouring postoperative endometrial cancer nodal volumes. *Int. J. Radiat. Oncol. Biol. Phys.* **79**(3), 943–947. 10.1016/j.ijrobp.2010.04.063 (2011). 21281897 10.1016/j.ijrobp.2010.04.063

[5] Sjöberg, C. *et al.* Clinical evaluation of multi-atlas based segmentation of lymph node regions in head and neck and prostate cancer patients. *Radiat. Oncol.* **8**, 229. 10.1186/1748-717X-8-229 (2013). 24090107 10.1186/1748-717X-8-229 PMC3842681

[6] Marschner, S., Datar, M., Gaasch, A., Xu, Z., Grbic, S., Chabin, G., Geiger, B., Rosenman, J., Corradini, S., Niyazi, M., Heimann, T., Möhler, C., Vega, F., Belka, C., Thieke, C. A deep image-to-image network organ segmentation algorithm for radiation treatment planning: principles and evaluation. Radiat. Oncol., 17(1):129. doi: 10.1186/s13014-022-02102-6 (2022). Erratum in: Radiat Oncol., 17(1):149 (2022). 10.1186/s13014-022-02102-6 PMC9308364 35869525

[7] Costea, M. *et al.* Comparison of atlas-based and deep learning methods for organs at risk delineation on head-and-neck CT images using an automated treatment planning system. *Radiother. Oncol.* **177**, 61–70. 10.1016/j.radonc.2022.10.029 (2022). 36328093 10.1016/j.radonc.2022.10.029

[8] van Dijk, L. V. *et al.* Improving automatic delineation for head and neck organs at risk by deep learning contouring. *Radiother. Oncol.* **142**, 115–123. 10.1016/j.radonc.2019.09.022 (2020). 31653573 10.1016/j.radonc.2019.09.022

[9] Urago, Y. *et al.* Evaluation of auto-segmentation accuracy of cloud-based artificial intelligence and atlas-based models. *Radiat. Oncol.* **16**(1), 175. 10.1186/s13014-021-01896-1 (2021). 34503533 10.1186/s13014-021-01896-1 PMC8427857

[10] Chen, X. *et al.* A deep learning-based auto-segmentation system for organs-at-risk on whole-body computed tomography images for radiation therapy. *Radiother. Oncol.* **160**, 175–184. 10.1016/j.radonc.2021.04.019 (2021). 33961914 10.1016/j.radonc.2021.04.019

[11] Choi, M. S. *et al.* Clinical evaluation of atlas- and deep learning-based automatic segmentation of multiple organs and clinical target volumes for breast cancer. *Radiother. Oncol.* **153**, 139–145. 10.1016/j.radonc.2020.09.045 (2020). 32991916 10.1016/j.radonc.2020.09.045

[12] Hui, C. B. *et al.* Quality assurance tool for organ at risk delineation in radiation therapy using a parametric statistical approach. *Med. Phys.* **45**(5), 2089–2096. 10.1002/mp.12835 (2018). 29481703 10.1002/mp.12835 PMC8220860

[13] Altman, M. B. *et al.* A framework for automated contour quality assurance in radiation therapy including adaptive techniques. *Phys. Med. Biol.* **60**(13), 5199–5209. 10.1088/0031-9155/60/13/5199 (2015). 26083863 10.1088/0031-9155/60/13/5199

[14] Zhang, Y., Plautz, T. E., Hao, Y., Kinchen, C. & Li, X. A. Texture-based, automatic contour validation for online adaptive replanning: A feasibility study on abdominal organs. *Med. Phys.* **46**(9), 4010–4020. 10.1002/mp.13697 (2019). 31274193 10.1002/mp.13697

[15] Nourzadeh, H. *et al.* Knowledge-based quality control of organ delineations in radiation therapy. *Med. Phys.***49**(3), 1368–1381. 10.1002/mp.15458 (2022).35028948 10.1002/mp.15458

[16] Duan, J. *et al.* Contouring quality assurance methodology based on multiple geometric features against deep learning auto-segmentation. *Med. Phys.* **50**(5), 2715–2732. 10.1002/mp.16299 (2023). 36788735 10.1002/mp.16299 PMC10175153

[17] Men, K., Geng, H., Biswas, T., Liao, Z. & Xiao, Y. Automated quality assurance of OAR contouring for lung cancer based on segmentation with deep active learning. *Front. Oncol.* **10**, 986. 10.3389/fonc.2020.00986 (2020). 32719742 10.3389/fonc.2020.00986 PMC7350536

[18] Chen, X. *et al.* CNN-based quality assurance for automatic segmentation of breast cancer in radiotherapy. *Front. Oncol.* **10**, 524. 10.3389/fonc.2020.00524 (2020). 32426272 10.3389/fonc.2020.00524 PMC7212344

[19] Rhee, D. J. *et al.* Automatic detection of contouring errors using convolutional neural networks. *Med. Phys.* **46**(11), 5086–5097. 10.1002/mp.13814 (2019). 31505046 10.1002/mp.13814 PMC6842055

[20] Piper, J., Nelson, A., Harper, J. Deformable image registration in MIM Maestro™ evaluation and description. MIM Software Inc. https://go.mimsoftware.com/hubfs/MimSoftware_September2020/pdf/Deformable_Image_Registration_in_MIM_Maestro_Evaluation_and_Description.pdf (2013).

[21] Wan, H. Automated contouring using neural networks. MIM Software Inc. https://go.mimsoftware.com/hubfs/5300642/TD650-Contour_ProtegeAI_White_Paper-20211208.pdf (2020).

[22] Taha, A. A. & Hanbury, A. Metrics for evaluating 3D medical image segmentation: Analysis, selection, and tool. *BMC Med. Imaging* **15**, 29. 10.1186/s12880-015-0068-x (2015). 26263899 10.1186/s12880-015-0068-x PMC4533825

